# Bringing the age-related macular degeneration high-risk allele age-related maculopathy susceptibility 2 into focus with stem cell technology

**DOI:** 10.1186/s13287-017-0584-4

**Published:** 2017-06-06

**Authors:** Shuo Sun, ZhiQing Li, Patrick Glencer, BinCui Cai, XiaoMin Zhang, Jin Yang, XiaoRong Li

**Affiliations:** 1 Tianjin Medical University Eye Hospital, Tianjin, 300384 China; 2 Nova Southeastern College of Optometry, Fort Lauderdale, FL 33314 USA

**Keywords:** AMD, *ARMS2*, RPE cells, Stem cells, Genetic model

## Abstract

Age-related macular degeneration (AMD) is a major cause of blindness in older adults in developed countries. It is a multifactorial disease triggered by both environmental and genetic factors. High-temperature requirement A serine peptidase 1 (*HTRA1*) and age-related maculopathy susceptibility 2 (*ARMS2*) are two genes that are strongly associated with AMD. Because *ARMS2* is an evolutionarily recent primate-specific gene and because the *ARMS2*/*HTRA1* genes are positioned at a locus on chromosome 10q26 in a region with strong linkage disequilibrium, it is difficult to distinguish the functions of the individual genes. Therefore, it is necessary to bring these genes into focus. Patient-specific induced pluripotent stem cell (iPSC)-derived retinal pigment epithelium (RPE) provides direct access to a patient’s genetics and allows for the possibility of identifying the initiating events of RPE-associated degenerative diseases. In this paper, a review of recent epidemiological studies of AMD is offered. An argument for a definite correlation between the *ARMS2* gene and AMD is presented. A summary of the use of *ARMS2* genotyping for medical treatment is provided. Several *ARMS2-*related genetic models based on such stem cells as iPSCs are introduced. The possibility of applying gene-editing techniques and stem-cell techniques to better explore the mechanisms of the *ARMS2* high-risk allele, which will lead to important guidance for treatment, is also discussed.

## Background

Age-related macular degeneration (AMD), also known as senile macular degeneration, is one of the primary causes of irreversible visual impairment among the elderly in the developed world [1, 2]. The incidence of AMD among Asian adults over the age of 50 is high [3, 4]. AMD has increasingly become a global issue. Several population-based studies have examined AMD worldwide [5–9]. Although the data are different in each study due to racial differences and participant recruitment, the obvious tendency towards an increased prevalence cannot be ignored.

AMD is a multifactorial disease due to interactions between genetic and environmental risk factors [10]. Genome-wide association studies revealed that variations in or near the complement factor H (*CFH*), complement factor I (*CFI*), complement factor B (*CFB*), and complement 3 (*C3*) genes are significantly associated with AMD. In addition to mutations in complement genes, a polymorphism (rs10490924) in *ARMS2* (age-related maculopathy susceptibility 2), which encodes a protein that binds to the cell surface and enhances complement activation, shows the strongest association with AMD [11]. Several challenges remain regarding *ARMS2*. First, the cellular expression and function of *ARMS2* in AMD are not fully understood. Second, whether the efficacy of medical treatment has a relationship with risk alleles remains controversial. Third, the *ARMS2* gene is present only in primates, which makes it difficult to construct animal models.

Given this situation and the rapid development of regenerative medical therapy, many researchers have performed deep and fruitful analyses of AMD and *ARMS2*. A recent report using a bisretinoid N-retinylidine-N-ethanolamine (A2E)-stressed human induced pluripotent stem cell (iPSC) model of AMD indicates that *ARMS2*/*HTRA1* compromises the superoxide dismutase 2 response, suggesting increased vulnerability to oxidative stress [12]. In 2016, Saini et al. used a human iPSC model of AMD to demonstrate that nicotinamide altered disease-related phenotypes (*ARMS2*/*HTRA1*) by inhibiting drusen proteins and inflammatory and complement factors [13]. With the help of stem cells or iPSC genetic models, researchers can better simulate the morbid state and then explore how the*ARMS2*/*HTRA1* genes affect AMD.

## *ARMS2* variants and AMD

Because *ARMS2* is an evolutionarily recent primate-specific gene and because the *ARMS2*/*HTRA1* genes are found at a locus on chromosome 10q26 in a region with strong linkage disequilibrium [14], whether *ARMS2* is the key gene leading to AMD is debatable.

Before we analyze the relationships between *ARMS2* polymorphisms and AMD, we must summarize the basic information available about *ARMS2* polymorphisms (Table 1). Recently, Grassmann et al. [15] demonstrated that genetic variants in or close to *ARMS2* but not *HTRA1* are responsible for disease susceptibility at the 10q26 locus. These results encourage a focus on functional analyses of *ARMS2* and its role in AMD pathogenesis [15], even though a study by Yang et al. [16] suggested that a variant of the *HTRA1* gene increased susceptibility to AMD. Jabbarpoor Bonyadi et al. considered that *ARMS2*/LOC387715 and *CFH* share a common pathway, possibly the complement system pathway, in AMD pathogenesis, and proposed that these genes might have a synergistic effect in AMD [17]. Furthermore, *R38X*, *A69S*, and *R3H* (rs10490923), which are the three common coding variants in the *ARMS2* gene, were tested in a large case-control data set; the analysis showed that the *A69S* variant was significantly associated with AMD risk. The effect of the *R3H* variant was the opposite after adjustment for sex and age. Wang et al. [18] showed that if the effect of *A69S* is removed, the inverse effect of *R3H* is no longer statistically significant, revealing the strong linkage disequilibrium in this region and showing that the *A69S* variant is a strong risk factor for AMD. Kortvely et al. [19] found chimeric transcripts of the *PLEKHA1* (pleckstrin homology domain-containing protein family A member 1) gene that ended in *ARMS2*, which may explain how variants in this locus affect AMD. From these studies, it may be concluded that a close relationship between *ARMS2* variations and AMD exists, but the specific mechanism remains to be confirmed.

**Table 1:** Basic information for single nucleotide polymorphisms of four susceptibility loci in *ARMS2*

SNP	Physical location	Function	Allele	Minor allele	Minor allele frequency
rs10490923	10:122454735	Intron variant, missense	A/G	A	0.022
rs10490924	10:122454932	Intron variant, missense	G/T	T	0.402
rs2736911	10:122454839	Intron variant, stop gained	C/T	T	0.122
rs2736912	10:122456078	Intron variant	C/T	T	0.081

## Medical treatment effects and AMD genetic variations

In recent years, the prevalence of AMD has increased, and medical treatment options are developing quickly [20]. The argument that it is necessary to perform genotyping during the medical treatment of AMD has garnered attention over the past few years. Awh et al. [21, 22] suggested that the treatment response to antioxidants and zinc differs based on various combinations of *CFH* risk alleles and *ARMS2* risk alleles, an idea that was inconsistent with a previous study by Lee and Brantley [23]. Chew et al. [24] opposed this idea as well, demonstrating that the genotypes at the *CFH* and *ARMS2* loci did not significantly alter the benefits of Age-Related Eye Disease Study (AREDS) supplements, including a placebo, antioxidants, zinc, or antioxidants plus zinc. Smailhodzic et al. [25] revealed that, although there was no significant association between changes in complement catabolism and *CFH* and *ARMS2* genotypes, there was evidence that the daily administration of 50 mg of zinc sulfate could inhibit complement catabolism in AMD patients with increased complement activation and slow AMD progression.

These findings raise concerns about genotyping during the medical treatment of AMD. To date, the results for AREDS supplement use have shown dissimilar effects with different genotypes (summarized in Table 2). Basic research is greatly needed to resolve the question of whether genotyping is important.

**Table 2:** Effects of AREDS supplements on various combinations of *CFH* and *ARMS2* genotypes

Author	Patient information	Genotype		AREDS supplement	Outcome measure	Result	Authors’ view with respect to genotyping utility
Awh et al. 2015 [21]	989 white patients with category 3 or 4 AMD	0 or 1 *CFH* risk alleles and no *ARMS2* risk alleles		Components of the AREDS formulation	Seven-year treatment-specific progression rates	Placebo,22.6%;with antioxidants,9.17% (*P* = 0.033)	Support
		0 or 1 *CFH* risk alleles and 1 or 2 *ARMS2* risk alleles				Placebo, 43.3%; zinc, 25.2% (*P* = 0.020); AREDS formulation (AF), 27.3% (*P* = 0.011)	
		2 *CFH* risk alleles and no *ARMS2* risk alleles				Placebo, 17.0%; zinc, 43.2% (*P* = 0.023); AF, 40.2% (*P* = 0.039)	
		2 *CFH* risk alleles and 1 or 2 *ARMS2* risk alleles				No treatment was better than placebo (48.4%)	
Lee et al. 2008 [23]	876 participants at high risk for developing advanced AMD	*CFH*	TT	Antioxidants + zinc, Antioxidants, Zinc	Progression from high risk to advanced AMD	There was a nominally statistically significant interaction between AREDS supplements and the *CFH* Y402H genotype, and no significant treatment interactions were observed with *ARMS2*	Reject
			TC				
			CC				
		*ARMS2*	GG				
			TG				
			TT				
Chew et al. 2014 [24]	1237 genotyped AREDS participants of Caucasian ethnicity who were at risk of developing late AMD	*CFH*/*ARMS2* = xy, where x = *CFH* risk group, and y = *ARMS2* risk alleles		Placebo, antioxidants (vitamins C, E, beta-carotene), zinc with copper, or a combination of antioxidants, zinc, and copper	Progression to late AMD	No clinically significant association between *CFH* and *ARMS2* genotypes and response to nutritional supplements	Reject
		*00*					
		*01*					
		*02*					
		*10*					
		*11*					
		*12*					
		*20*					
		*21*					
		*22*					
Smailhodzic et al. 2014 [25]	72 patients with various stages of AMD	*CFH*	TT	A daily supplement of 50 mg zinc sulfate and 1 mg cupric sulfate for 3 months	The effect of zinc on complement activation in vitro	There was no significant association between changes in complement catabolism and *CFH* and *ARMS2* genotypes, but AMD progression was slowed	Reject
			TC				
			CC				
		*ARMS2*	GG				
			TG				
			TT				

At present, vascular endothelial growth factor inhibitors (anti-VEGF) have become the first-line medication for the treatment of choroidal neovascularisation [26]. Ranibizumab is used frequently for neovascular AMD and for polypoidal choroidal vasculopathy. Hu et al. [27] indicated that, in patients with the *A69S* variant, the East Asian population showed better response to anti-angiogenic treatment than the Caucasian population on the basis of a meta-analysis. Piermarocchi et al. [28] and Park et al. [29] indicated that the TT genotype of *VEGFA* was associated with a significantly higher chance of visual gain than other genotypes. With respect to the tomographic outcome, the GG genotypes of both *ARMS2* and *HTRA1* were associated with larger central subfield macular thickness reduction than other genotypes, and no polymorphism showed a significant association with the frequency of injections. In contrast, complement factor H risk alleles affected the mid-term response to ranibizumab in exudative AMD, with worse 12-month best-corrected visual acuity (BCVA) outcomes, but there was no statistically significant association between *ARMS2* and the curative effect. Dedania et al. [30] reviewed 16 different types of studies with follow-up periods that assessed 18 different genes and found that the frequency of injections may be associated with certain genotypes and that different genotypes might affect an individual’s response to treatment for neovascular AMD.

## Stem cell technology and *ARMS2* research

### Stem cell transplantation as a potential treatment

Many different forms of blindness result from the dysfunction or loss of the outer retina, including AMD and hereditary retinal degeneration. Various genetic diseases, such as Best disease, retinitis pigmentosa (RP), and Stargardt disease (STGD), are increasingly becoming important causes of irreversible blindness. However, effective treatments for these diseases have not yet been developed. Therefore, cell replacement is becoming a new method of treatment for outer-retina diseases. Fortunately, the differentiation of retinal pigment epithelium (RPE) from human embryonic stem cells (hESCs), from mesenchymal stem cells (MSCs), and from iPSCs has created many potential ways to replace dead or dying RPE. These cells have been shown to merge into the retina and regain functionality [31].

hESC-RPE replacement therapy is one current research focus. Qu et al. [32] successfully differentiated rat ESCs into glia-enriched retinal progenitor cells (RPCs) and retinal neuron-enriched RPCs in vitro; these cells might be useful for the treatment of such degenerative retinal diseases as AMD, RP, and STGD. Schwartz et al. [33] investigated the primary safety and tolerability endpoints of the subretinal transplantation of hESC-derived RPE in nine patients with Stargardt’s macular dystrophy (aged >18 years) and nine with atrophic AMD (aged >55 years). This study provided the first evidence of the medium- to long-term safety, graft survival, and possible biological activity of pluripotent stem cell progeny in individuals with Stargardt’s macular dystrophy and AMD [33]. Buchholz et al. [34] described a new protocol that was useful for rapidly generating RPE for transplantation. Although RPE from hESCs is an ideal RPE source for patients with dry AMD, it has potential risks. Ethical issues and lifelong immune rejection limit its application for modeling and transplant therapy in the clinical setting.

Recently, two encouraging reports using a rat model showed that erythropoietin (EPO) gene modification in MSCs may represent an even better source of RPE [35, 36]. As the derivation of RPE from MSCs is a new method for transplantation, further research in animal models is required to verify these results.

iPSCs, which originate from somatic cells, bear patient-specific genetic or protein information that may have potential for use in the development of personalized therapeutic approaches. Silva et al. [37] proposed standardized practices for iPSC generation and analyzed bottlenecks and future directions of this technique. Gong et al. [38] found that protein-induced pluripotent stem cells can be differentiated into RPE-like cells using a method that shows an increased safety profile, a critical consideration for the development of better treatments for degenerative retinal diseases such as AMD. Mandai et al. [39] demonstrated the feasibility of transplanting a sheet of RPE cells differentiated from iPSCs into a patient with neovascular AMD one year after surgery. The transplanted cell sheet remained intact; however, the BCVA neither improved nor worsened, and Central Macular Edema (CME) was present [39]. Although iPSCs have enormous potential for clinical applications, they also present several challenges. For example, they are derived from the patients themselves and thus may carry disease-causing genes, and they pose a potential risk of carcinoma [40, 41]. In future iPSC research, investigators should conduct detailed studies that focus on high-yield components to better apply iPSCs to humans.

### *ARMS2* research based on the iPSC-RPE AMD model

The development of iPSC technology has caused a stunning shift in the field of stem cell biology and has provided an alternative source of pluripotent cells [42]. The pioneering accomplishment in 2006 by the Yamanaka group had important implications for the field [43]. Several retinal models have been developed. Singh et al. [44] used Best patient-specific iPSC-derived RPE to simulate some of the cellular disease endophenotypes in RPE cells; Megaw et al. [45] used human iPSCs with RP-causing mutations to examine the pathophysiology of disease; and Yvon et al. [46] published a comprehensive paper about using stem cells to model diseases of the outer retina.

Genome-wide association studies have identified risk alleles for the disease, such as those of the *ARMS2* and *HTRA1* genes, but how these alleles lead to pathology remains unclear. There is currently a dearth of appropriate models for AMD; autopsied eyes from end-stage patients already possess terminal changes and therefore cannot be used to determine how abnormal gene expression can lead to RPE pathology, and mice do not have maculae [47]. As noted above, iPSC-RPE derived from AMD patients can model aspects of AMD in culture and is a better model than RPE stem cells. There is ample opportunity to study the *ARMS2* gene using iPSC-RPE cells that are derived from AMD patients themselves and express multiple biomarkers associated with AMD and AMD-associated proteins.

Superoxide dismutase 2 (SOD2), encoded by the *SOD2* gene, is a mitochondrial enzyme that protects cells from oxidative damage [48]. Yang et al. [12] created a model of AMD by obtaining patient-specific iPSC-derived RPE and pharmacologically accelerating the ageing process by treatment with A2E and blue light. They found with this iPSC-RPE AMD model that the *ARMS2*/*HTRA1* risk alleles decreased SOD2 defense, making RPE more susceptible to oxidative damage and thereby contributing to AMD pathogenesis [12]. However, these findings would be better supported if they were reproduced using additional patient samples with identical *ARMS2*/*HTRA1* risk alleles. Using this ground-breaking iPSC model, researchers can better assess the effects of *ARMS2*/*HTRA1* risk alleles on AMD pathogenesis and identify pathways that can be therapeutically targeted. Furthermore, Saini et al. [13] revealed the function of nicotinamide in AMD using the iPSC model; they noted that ICAM1, which has been linked to the development of the wet form of AMD, was strongly upregulated in human AMD *ARMS2*/*HTRA1* iPSC-RPE and was markedly inhibited by nicotinamide [13].

Due to the 100% linkage disequilibrium between the*ARMS2* and *HTRA1* risk alleles, it has not been possible to establish which of these genes is the causal variant for AMD. Fortunately, in 2013, two American laboratories discovered that the clustered regularly interspaced short palindromic repeats/Cas-9(CRISPR/Cas9) system of prokaryotic adaptive immunity might be used as a new method for genome editing [49]. This technique may provide insight into iPSC-RPE AMD modeling. Researchers can now edit the genotypes at will, and the resulting iPSC-RPE cells can serve as models of specific *ARMS2* risk alleles. This strategy is similar in principle to deriving iPSCs from patients with identical *ARMS2*/*HTRA1* risk alleles. Furthermore, the genome-editing model has overtaken models using patient-specific hESCs due to the ethical, technical, and political concerns associated with such cells [50].

## Conclusions

For the purposes discussed above, further study is needed for a greater understanding of the characteristics of *ARMS2* gene-related AMD modeled using stem cell technology. The identification of additional disease-specific biological characteristics and a fuller understanding of the mechanisms of induction and migration will make drug discovery and cell therapy gradually change from dream to reality. Although they remain difficult to apply to clinical practice, several researchers have found that iPSCs have great potential for regenerative medicine. Continuing the progress of cell transplantation and regeneration in the retina and in AMD genetic models will prove to be beneficial. Regenerative medicine in the eye must be further improved so that it can be more effectively employed.

Although several issues remain to be solved, the findings discussed above provide new ideas for continuing exploration of the mechanisms by which *ARMS2* high-risk alleles lead to AMD; such research will provide important guidance for the treatment of AMD.

## Abbreviations

A2E: Bisretinoid N-retinylidine-N-ethanolamine
AMD: Age-related macular degeneration
AREDS: Age-Related Eye Disease Study
ARMS2: Age-related maculopathy susceptibility 2
BCVA: Best corrected visual acuity
hESC: Human embryonic stem cell
HTRA1: High-temperature requirement A serine peptidase 1
iPSC: Induced pluripotent stem cell
MSC: Mesenchymal stem cell
RP: Retinitis pigmentosa
RPC: Retinal progenitor cell
RPE: Retinal pigment epithelium
SNP: Single nucleotide polymorphism
SOD2: Superoxide dismutase 2
STGD: Stargardt disease

## References

[1] Owen CG, Jarrar Z, Wormald R, Cook DG, Fletcher AE, Rudnicka AR (2012). The estimated prevalence and incidence of late stage age related macular degeneration in the UK. Br J Ophthalmol..

[2] Krause L, Yousif T, Pohl K, O.B.O.C.S. Group (2013). An epidemiological study of neovascular age-related macular degeneration in Germany. Curr Med Res Opin.

[3] Cheung CM, Li X, Cheng CY, Zheng Y, Mitchell P, Wang JJ, Wong TY (2014). Prevalence, racial variations, and risk factors of age-related macular degeneration in Singaporean Chinese, Indians, and Malays. Ophthalmology..

[4] You QS, Xu L, Yang H, Li YB, Wang S, Wang JD, Zhang JS, Wang YX, Jonas JB (2012). Five-year incidence of age-related macular degeneration: the Beijing Eye Study. Ophthalmology..

[5] Cheng CY, Yamashiro K, Chen LJ, Ahn J, Huang L, Huang L, Cheung CMG, Miyake M, Cackett PD, Yeo IY (2015). New loci and coding variants confer risk for age-related macular degeneration in East Asians. Nat Commun..

[6] Wang IK, Lin HJ, Wan L, Lin CL, Yen TH, Sung FC (2016). Risk of age-related macular degeneration in end-stage renal disease patients receiving long-term dialysis. Retina..

[7] Park SJ, Kwon KE, Choi NK, Park KH, Woo SJ (2015). Prevalence and incidence of exudative age-related macular degeneration in South Korea: a nationwide population-based study. Ophthalmology..

[8] Friedman DS, O'Colmain BJ, Muñoz B, Tomany SC, McCarty C, de Jong PT, Nemesure B, Mitchell P, Kempen J, Eye Diseases Prevalence Research Group (2004). Prevalence of age-related macular degeneration in the United States. Arch Ophthalmol.

[9] Mukesh BN, Dimitrov PN, Leikin S, Wang JJ, Mitchell P, McCarty CA, Taylor HR (2004). Five-year incidence of age-related maculopathy: the Visual Impairment Project. Ophthalmology.

[10] Armstrong RA, Mousavi M (2015). Overview of risk factors for age-related macular degeneration (AMD). J Stem Cells.

[11] Micklisch S, Lin Y, Jacob S, Karlstetter M, Dannhausen K, Dasari P, von der Heide M, Dahse HM, Schmölz L, Grassmann F (2017). Age-related macular degeneration associated polymorphism rs10490924 in ARMS2 results in deficiency of a complement activator. J Neuroinflammation..

[12] Yang J, Li Y, Chan L, Tsai YT, Wu WH, Nguyen HV, Hsu CW, Li X, Brown LM, Egli D (2014). Validation of genome-wide association study (GWAS)-identified disease risk alleles with patient-specific stem cell lines. Hum Mol Genet..

[13] Saini JS, Corneo B, Miller JD, Kiehl TR, Wang Q, Boles NC, Blenkinsop TA, Stern JH, Temple S (2017). Nicotinamide ameliorates disease phenotypes in a human iPSC model of age-related macular degeneration. Cell Stem cell.

[14] Wang G (2014). Chromosome 10q26 locus and age-related macular degeneration: a progress update. Exp Eye Res.

[15] Grassmann F, Heid IM, Weber BHF (2017). Recombinant haplotypes narrow the ARMS2/HTRA1 association signal for age-related macular degeneration. Genetics.

[16] Yang Z, Camp NJ, Sun H, Tong Z, Gibbs D, Cameron DJ, Chen H, Zhao Y, Pearson E, Li X (2006). A variant of the HTRA1 gene increases susceptibility to age-related macular degeneration. Science..

[17] Jabbarpoor Bonyadi MH, Yaseri M, Bonyadi M, Soheilian M, Karimi S (2016). Association of combined complement factor H Y402H and ARMS/LOC387715 A69S polymorphisms with age-related macular degeneration: a meta-analysis. Curr Eye Res..

[18] Wang G, Scott WK, Agarwal A, Haines JL, Pericakvance MA (2013). Coding variants in ARMS2 and the risk of age-related macular degeneration. JAMA Ophthalmol..

[19] Kortvely E, Ueffing M (2016). Gene structure of the 10q26 locus: a clue to cracking the ARMS2/HTRA1 riddle?. Adv Exp Med Biol..

[20] Yonekawa Y, Kim IK (2014). Clinical characteristics and current treatment of age-related macular degeneration. Cold Spring Harbor Perspect Med.

[21] Awh CC, Hawken S, Zanke BW (2015). Treatment response to antioxidants and zinc based on CFH and ARMS2 genetic risk allele number in the Age-Related Eye Disease Study. Ophthalmology..

[22] Awh CC, Lane AM, Hawken S, Zanke B, Kim IK (2013). CFH and ARMS2 genetic polymorphisms predict response to antioxidants and zinc in patients with age-related macular degeneration. Ophthalmology.

[23] Lee AY, Brantley MA (2008). CFH and LOC387715/ARMS2 genotypes and antioxidants and zinc therapy for age-related macular degeneration. Pharmacogenomics..

[24] Chew EY, Klein ML, Clemons TE, Agrón E, Ratnapriya R, Edwards AO, Fritsche LG, Swaroop A, Abecasis GR (2014). No clinically significant association between CFH and ARMS2 genotypes and response to nutritional supplements: AREDS Report Number 38. Ophthalmology..

[25] Smailhodzic D, Asten FV, Blom AM, Mohlin FC, Hollander AID, van de Ven JPH, Huet RACV, Groenewoud JMM, Tian Y, Berendschot TTJM (2014). Zinc supplementation inhibits complement activation in age-related macular degeneration. Plos One..

[26] Johnston SS, Wilson K, Huang A, Smith D, Varker H, Turpcu A (2013). Retrospective analysis of first-line anti-vascular endothelial growth factor treatment patterns in wet age-related macular degeneration. Adv Ther..

[27] Hu Z, Xie P, Ding Y, Yuan D, Liu Q (2014). Association between variants A69S in ARMS2 gene and response to treatment of exudative AMD: a meta-analysis. Br J Ophthalmol..

[28] Piermarocchi S, Miotto S, Colavito D, Leon A, Segato T (2014). Combined effects of genetic and non‐genetic risk factors affect response to ranibizumab in exudative age‐related macular degeneration. Acta Ophthalmol..

[29] Park UC, Shin JY, McCarthy LC, Kim SJ, Park JH, Chung H, Yu HG (2014). Pharmacogenetic associations with long-term response to anti-vascular endothelial growth factor treatment in neovascular AMD patients. Mol Vis..

[30] Dedania VS, Grob S, Zhang K, Bakri SJ (2015). Pharmacogenomics of response to anti-VEGF therapy in exudative age-related macular degeneration. Retina..

[31] Nazari H, Zhang L, Zhu D, Chader GJ, Falabella P, Stefanini F, Rowland T, Clegg DO, Kashani AH, Hinton DR (2015). Stem cell based therapies for age-related macular degeneration: The promises and the challenges. Prog Retin Eye Res..

[32] Qu Z, Guan Y, Cui L, Song J, Gu J, Zhao H, Xu L, Lu L, Jin Y, Xu GT (2015). Transplantation of rat embryonic stem cell-derived retinal progenitor cells preserves the retinal structure and function in rat retinal degeneration. Stem Cell Res Ther..

[33] Schwartz SD, Regillo CD, Lam BL, Eliott D, Rosenfeld PJ, Gregori NZ, Hubschman JP, Davis JL, Heilwell G, Spirn M (2015). Human embryonic stem cell-derived retinal pigment epithelium in patients with age-related macular degeneration and Stargardt’s macular dystrophy: follow-up of two open-label phase 1/2 studies. Lancet..

[34] Buchholz DE, Pennington BO, Croze RH, Hinman CR, Coffey PJ, Clegg DO (2013). Rapid and efficient directed differentiation of human pluripotent stem cells into retinal pigmented epithelium. Stem Cells Transl Med.

[35] Guan Y, Cui L, Qu Z, Lu L, Wang F, Wu Y, Zhang J, Gao F, Tian H, Xu L (2013). Subretinal transplantation of rat MSCs and erythropoietin gene modified rat MSCs for protecting and rescuing degenerative retina in rats. Curr Mol Med..

[36] Tzameret A, Sher I, Belkin M, Treves AJ, Meir A, Nagler A, Levkovitchverbin H, Barshack I, Rosner M, Rotenstreich Y (2014). Transplantation of human bone marrow mesenchymal stem cells as a thin subretinal layer ameliorates retinal degeneration in a rat model of retinal dystrophy. Exp Eye Res..

[37] Silva M, Daheron L, Hurley H (2015). Generating iPSCs: translating cell reprogramming science into scalable and robust biomanufacturing strategies. Cell Stem Cell.

[38] Gong J, Fields MA, Moreira EF, Bowrey HE, Gooz M, Ablonczy Z, Priore LVD (2015). Differentiation of human protein-induced pluripotent stem cells toward a retinal pigment epithelial cell fate. PLoS One..

[39] Mandai M, Watanabe A, Kurimoto Y, Hirami Y, Morinaga C, Daimon T, Fujihara M, Akimaru H, Sakai N, Shibata Y, Terada M, Nomiya Y, Tanishima S, Nakamura M, Kamao H, Sugita S, Onishi A, Ito T, Fujita K, Kawamata S, Go MJ, Shinohara C, Hata KI, Sawada M, Yamamoto M, Ohta S, Ohara Y, Yoshida K, Kuwahara J, Kitano Y, Amano N, Umekage M, Kitaoka F, Tanaka A, Okada C, Takasu N, Ogawa S, Yamanaka S, Takahashi M (2017). Autologous induced stem-cell-derived retinal cells for macular degeneration. N Engl J Med.

[40] Nori S, Okada Y, Nishimura S (2015). Long-term safety issues of iPSC-based cell therapy in a spinal cord injury model: oncogenic transformation with epithelial-mesenchymal transition. Stem Cell Rep.

[41] Seminatore C, Polentes J, Ellman D, Kozubenko N, Itier V, Tine S, Tritschler L, Brenot M, Guidou E, Blondeau J, Lhuillier M, Bugi A, Aubry L, Jendelova P, Sykova E, Perrier AL, Finsen B, Onteniente B (2010). The postischemic environment differentially impacts teratoma or tumor formation after transplantation of human embryonic stem cell-derived neural progenitors. Stroke.

[42] Fields M, Cai H, Gong J, Priore LD (2016). Potential of induced pluripotent stem cells (iPSCs) for treating age-related macular degeneration (AMD). Cells..

[43] Takahashi K, Yamanaka S. Induction of pluripotent stem cells from mouse embryonic and adult fibroblast cultures by defined factors. Cell. 2006;126(4):663–76. 10.1016/j.cell.2006.07.024 16904174

[44] Singh R, Shen W, Kuai D, Martin JM, Guo X, Smith MA, Perez ET, Phillips MJ, Simonett JM, Wallace KA, Verhoeven AD, Capowski EE, Zhang X, Yin Y, Halbach PJ, Fishman GA, Wright LS, Pattnaik BR, Gamm DM (2013). iPS cell modeling of Best disease: insights into the pathophysiology of an inherited macular degeneration. Hum Mol Genet..

[45] Megaw R, Mellough C, Wright A, Lako M, Ffrench-Constant C (2015). Use of induced pluripotent stem-cell technology to understand photoreceptor cytoskeletal dynamics in retinitis pigmentosa. Lancet.

[46] Yvon C, Ramsden CM, Lane A, Powner MB, da Cruz L, Coffey PJ, Carr AJ (2015). Using stem cells to model diseases of the outer retina. Comput Struct Biotechnol J.

[47] Nguyen HV, Li Y, Tsang SH (2015). Patient-specific iPSC-derived RPE for modeling of retinal diseases. J Clin Med..

[48] Cramer-Morales K, Heer CD, Mapuskar KA, Domann FE (2015). SOD2 targeted gene editing by CRISPR/Cas9 yields human cells devoid of MnSOD. Free Radic Biol Med..

[49] Doudna JA, Charpentier E (2014). Genome editing. The new frontier of genome engineering with CRISPR-Cas9. Science.

[50] Li Y, Chan L, Nguyen HV, Tsang SH (2016). Personalized medicine: cell and gene therapy based on patient-specific iPSC-derived retinal pigment epithelium cells. Adv Exp Med Biol..

